# Diabetes Self-Management Education in the Home

**DOI:** 10.7759/cureus.710

**Published:** 2016-07-25

**Authors:** Dianne Lavelle, Joanah Zeitoun, Marianne Stern, Elise Butkiewicz, Elfie Wegner, Courtney Reinisch

**Affiliations:** 1 School of Nursing, Rutgers University; 2 Overlook Family Medicine Residency, Atlantic Health

**Keywords:** chronic disease management, diabetes, home visit, home-based, dsme, diabetes self-management education

## Abstract

**Purpose:**

Diabetes self-management education and home visits have been found to improve clinical outcomes in individuals living with diabetes. The purpose of this pilot project was to evaluate the feasibility and effectiveness of conducting self-management education in patients' homes.

**Methods:**

Baseline biometric data was collected from a cohort of adult patients with diabetes. Home visits to 19 patients were conducted by doctoral students from Rutgers University School of Nursing. The visits included knowledge assessment, review of foods in the home, diabetes self-management education, and teaching the proper use of monitoring tools such as the glucometer and blood pressure monitor. Biomarkers were obtained post-intervention and were compared to baseline biomarkers. Descriptive lifestyle data was collected and opportunities for customized patient education were provided.

**Results:**

The biomarkers improved overall during the four months after the education intervention. The mean A1C reduced 12% (p=0.0107), the mean glucose reduced 12% (p=0.0994), the mean BMI reduced 2% (p=0.1490), the systolic pressure reduced 1% (p=0.4196), and the diastolic pressure remained stable. Specific goal setting further increased the improvement in the area the individual planned to address.

**Conclusions:**

This project supports prior studies that found that in-home educational programs can improve the self-management of diabetes and lead to improvement in health indicators. The benefits of the study included personal attention in ensuring the correct use of home health monitoring devices, building self-management confidence, and identifying treatment barriers that may not be easily discerned in a clinic setting.

## Introduction

­­­­­­

Diabetes is a chronic condition that can result in costly health-related complications, reduced quality of life, and loss of life. In the most recent national assessment of diabetes in the United States, the prevalence of diabetes was 9.3% with costs of diagnosed diabetes reaching $245 billion annually [1]. The need for individual self-management and patient participation in care is paramount. What makes diabetes unique is the need for sufficient self-management skills, which can result in improved quality of life and care [2]. Wakefield, et al. state that, “Transmission of education and advice to the patient on an ongoing basis with close surveillance by nurses can improve clinical outcomes in patients with comorbid chronic illness.” [3].

Diabetes Self-Management Education (DSME) has been found to improve biometrics in diabetic patients. In multiple studies, including research by Yuan, et al., Tshiananga, et al., Brunisholz, et al., Atak, et al., and Wattana, et al., DSME was found to effectively improve clinical markers such as A1C, glucose level, blood pressure, weight, lipids, and self-efficacy scores [4-8].

In studies by Ribeiro, et al., Cooper, et al., and others​ [9-10], in-home visits to patients for disease self-management, not necessarily diabetes self-management, have been found to improve clinical markers such as blood pressure, fasting glucose, body mass index (BMI), and self-efficacy score in patients with a chronic disease.

This project combines and interweaves two concepts: 1) the concept of DSME, which is not typically provided in a patient’s home, and 2) the concept of providing education in the home. In 2015, McElfish, et al. found that DSME in the home was effective when DSME was provided in a family setting in the home [11], but there is limited literature on this combined approach.

The purpose of this project was to examine whether in-home delivery of DSME to individuals with diabetes improves blood pressure, serum glucose, A1C, and BMI. DSME is typically delivered in a group or clinic setting. The intent of this project was to deliver the education at the patient’s place of residence, and evaluate the feasibility and effectiveness of this venue.

## Materials and methods

### Design

This project involved a cohort of adult diabetic patients. Baseline information was collected on A1C, fasting glucose, BMI, blood pressure, demographic information, and individual treatment goal. Home visits were conducted with all participants to augment services received at Overlook Family Medicine. The educational home visits were conducted by doctoral students from the School of Nursing, Rutgers University. The visits included knowledge assessment, DSME, review of food in the home, teaching proper use of home monitoring tools such as the glucometer and blood pressure monitors, and recording vital signs. In order to facilitate proper self-management behavior, participants were provided with free blood pressure monitors. The monitors were funded through a Novo Nordisk grant distributed by the Overlook Foundation through Overlook Family Medicine.

This study followed the cohort from the time of the in-home intervention until the time of the collection of post-intervention biomarkers at their next routine office visit. The next routine office visit occurred two to four months after the intervention. Participants were excluded from the study if the post-intervention biomarkers were not completed within four months after the intervention.

IRB approval was obtained from both Atlantic Health IRB and Rutgers University IRB before the project commenced. All patients provided written informed consent prior to participating and receiving the in-home visit.

### Participants

The target population comprised ambulatory patients with diabetes, who receive medical care at Overlook Family Medicine. A cohort of 19 patients was recruited from a diabetes group visit at the office and from among a random selection of patients on the office’s diabetes registry. The cohort comprised 12 females and seven males. The cohort included five Hispanic, seven African American, three Caucasian, and four Asian/other participants. The patients were between 40 and 90 years of age, with a mean age of 62 years.

### Intervention

At first contact with the participant, the principal investigator explained the purpose of the educational encounters and obtained the subject’s consent for in-home education, prior to commencing the educational encounter. All participants in the project received information on target goals for blood pressure, A1C, glucose, and BMI based on the American Diabetes Association (ADA) guidelines. The goals were individualized and determined at the group visit or during the in-home visit for non-group participants. All participants were provided free blood pressure monitors to facilitate self-management behaviors.

Each participant received one visit in their home and an additional visit if he/she did not show proper use of monitoring equipment at the first visit. Teaching tools were utilized during the visits to ensure consistency of self-monitoring steps. The patients were assessed for proper handling and use of medical supplies such as the blood pressure monitor, glucometer, and scale. During the home visit, the teaching adhered to self-monitoring steps from the American Heart Association for blood pressure self-monitoring [12]. For glucose self-monitoring, the teaching adhered to the ADA self-monitoring steps along with additional self-monitoring tips from the American College of Cardiology [13-14]. For BMI self-monitoring, the teaching utilized the BMI table from the National Institutes of Health [15].

During the in-home visits, the patients were assessed for any in-home barriers to achievement of their target goals. This descriptive data, such as the presence of cases of soda in the home, was collected for subsequent analysis. This data promoted opportunities for customized patient education.

In addition to the home visits, three follow-up telephone encounters were conducted. The follow-up phone calls were made during the two months after the home visit, during the second, fourth, and eighth week. The follow-up phone calls addressed any issues the patients were encountering, answered any questions the patients had, and helped to resolve any difficulties with self-monitoring of blood pressure, glucose, or BMI.

After the intervention, the electronic medical records were reviewed for A1C, fasting glucose, BMI, blood pressure and compared to the baseline to assess the effectiveness of the self-management intervention.

### Data storage

All underlying data for this study was stored, and continues to be stored, in patient charts in the secure McKesson's Horizon Ambulatory Care EMR in use by Overlook Family Medicine. These records remain available for additional analyses.

### Data analysis  

The group means were calculated on pre- and post-intervention data for each of the five biomarkers (A1C, fasting glucose, systolic pressure, diastolic pressure, BMI). The calculation of p values was conducted using one-tailed t-test for pre- and post-intervention for each of the five biomarkers. The pre-intervention biomarkers were excluded from the mean and t-test calculations when a patient’s post-intervention biomarker was not completed by four months following the intervention. All calculations were completed using Microsoft Excel. Guidance was received from Miguel Martinez, Institutional Research Specialist, Rutgers University, School of Nursing.

## Results

### Participation​

There were a total of 19 participants in the project. Each of the 19 participants received in-home DSME. Fourteen of the 19 participants completed all or some follow-up biomarker monitoring, either at Overlook Family Medicine or at an external laboratory. This equates to a 74% retention rate during the four-month study interval. Of the 14 retained, 13 participants went to a subsequent in-office appointment at Overlook Family Medicine where BMI and blood pressure were obtained, and most completed a follow-up laboratory monitoring including A1C and/or serum glucose at that time. One patient went to another facility for subsequent laboratory monitoring and her post-intervention laboratory findings were included in the results.

### Biomarker data

**Figure 1:**
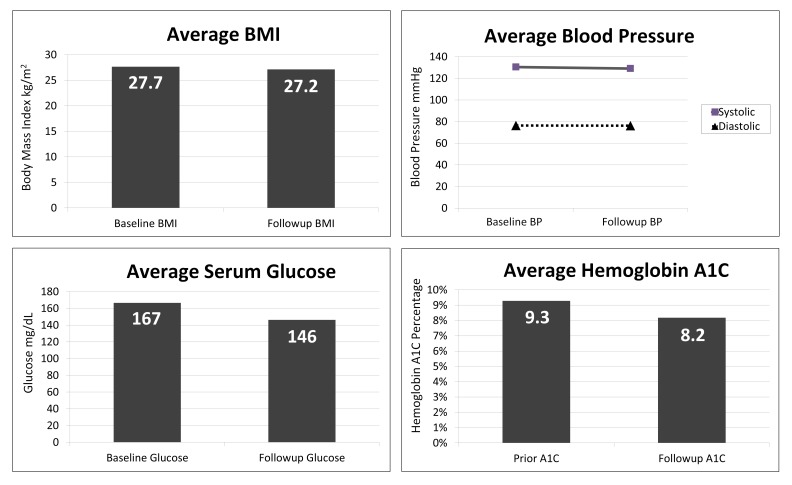
Comparison of pre- and post-intervention biomarkers shows improvement in BMI, serum glucose, and A1C.

The overall results demonstrate improved biomarkers after the education intervention as seen in Figure 1. The mean A1C reduced 12% relative from baseline, with an absolute reduction of 1.1% from 9.3% (78 mmol/mol) to 8.2% (66 mmol/mol) (p=0.0107). Ninety percent of the participants improved their A1C. Prior to the intervention, the most recent A1C of all participants was above 7% (53 mmol/mol), with two participants achieving an A1C below 7% (53 mmol/mol) after the intervention. One of the participants achieved an A1C of 6.5% (48 mmol/mol) after the intervention. No participants achieved an A1C below 6.5% (48 mmol/mol) after the intervention.

The mean serum glucose reduced 12% from 166.6 mg/dL (9.25 mmol/L) to 146.2 mg/dL (8.11 mmol/L) (p=0.0994). Seventy percent of the participants improved their serum glucose. At baseline, two participants had fasting glucose above 300 mg/dL (16.65 mmol/L), but no one was above 300 mg/dL post-intervention.

The mean BMI reduced 2% from 27.7 to 27.2 kg/m^2^ (p=0.1490). At baseline, 23% of the participants were classified as overweight (BMI 25 to 29.9 kg/m^2^), and 38% of the patients were classified as obese (BMI 30 kg/m^2^ or greater). Thirty-eight percent of the participants were normal weight at baseline. At post-intervention follow-up, 31% of the participants were classified as overweight, 31% of the participants were classified as obese, and 38% were normal weight. Forty-six percent of the participants exhibited a decrease in weight over the study period.

The mean blood pressure remained virtually constant as the mean systolic pressure reduced 1% from 130.5 mmHg to 129.7 mmHg (p=0.4196) and the mean diastolic pressure remained stable at 76.3 mmHg (p=0.4924). Prior to the intervention, 36% of the participants had a systolic pressure greater than or equal to 140 mmHg and 14% of the participants had a diastolic pressure greater than or equal to 90 mmHg. Following the intervention, 21% of the participants exhibited a systolic pressure greater than or equal to 140 mmHg and 7% of the participants exhibited a diastolic pressure greater than or equal to 90 mmHg. Altogether, 43% of the participants improved their systolic pressure and an equal number of the participants (43%) improved their diastolic pressure.

**Figure 2:**
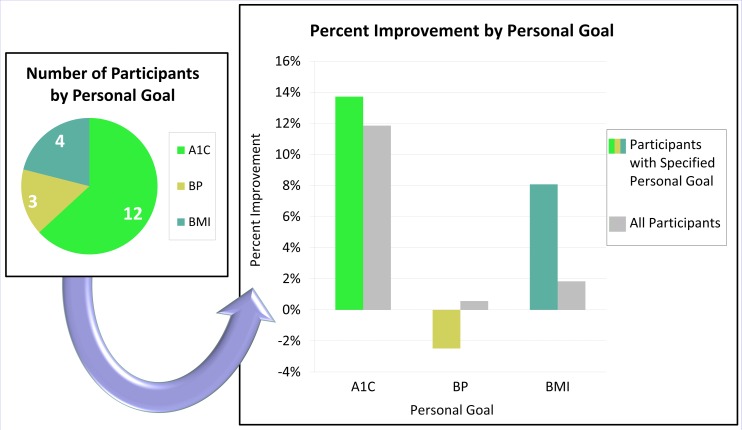
Greater improvements were seen with individualized goals if the participant specified a primary goal of improving their A1C or BMI.

Specific goal setting correlated with increased improvement in the area the individual planned to address (see Figure 2). The participants with a primary goal of reducing BMI achieved more improvement in BMI compared to all participants. A1C results followed this pattern but to a lesser degree. Blood pressure results did not follow this pattern. One participant, who aimed to reduce blood pressure, showed a significant systolic pressure increase of 46 mmHg over the baseline.

### Descriptive data

In addition to quantitative clinical data obtained through this project, descriptive data that would not be elicited during an in-office visit was gathered during the in-home visits. The list of descriptive data (see Table 1) included barriers in the home such as unhealthy foods and expired glucose test strips.

**Table 1:** Descriptive data was collected and opportunities for customized patient education were provided.

Care-Relevant Information:	Frequency:
Limited access to a kitchen	3
Mostly low glycemic foods in the home	7
Mostly high glycemic foods in the home	9
Lack of food in home	2
Cases of soda in the home	4
Positive in-home family support	13
No in-home family support	6
Expired glucose test strips	2
Improper use of glucometer	3
Uses body weight scale in home	12
(n=19)	

## Discussion

### Prior studies 

The results of this project agree directionally with prior studies conducted by Yuan, et al., Tshiananga, et al., and Brunisholz, et al. that found absolute changes of -0.20, -0.70, and -1.36 respectively in mean A1C after DSME [4-6]. This project found a post-DSME absolute change of -1.1 in mean A1C, falling within the mean effect range of these prior studies. 

The results also agree with studies by Ribeiro, et al. and Cooper, et al., that both found improved clinical markers after in-home visits that provided chronic disease education [9-10]. The aspects of improvement differ in this present project compared to these prior studies. This project yielded a similar improvement in serum glucose of -20 vs -19 as found by Ribeiro, et al. This project yielded a similar improvement in BMI of -0.5 vs -0.7 as in the Ribeiro, et al. study. This project found virtually no change in mean blood pressure although Ribeiro, et al. and Cooper, et al. saw mean systolic mmHg reductions of -13 and -15 in their studies respectively. The comparable in-home intervention studies by Ribeiro, et al. and Cooper, et al. did not measure A1C. Cooper, et al. did not measure glucose or BMI.

McElfish, et al. looked at the effect on A1C after DSME intervention in the family setting inside the patient’s home or church. At follow-up, they found an A1C change of -0.4, equating to a 5% improvement, compared to this present project that found an A1C change of -1.1, equating to a 12% improvement. However, it is important to note that their follow-up occurred at one month after the intervention and this study’s follow-up occurred two to four months after the intervention [11].

### Goal setting

Individual clinical indicators improved more when a participant had selected a goal for that specific indicator. The most commonly set goal was improvement of A1C followed by BMI and blood pressure. More participants improved A1C and glucose compared to any other indicator, likely due to a large percent of participants aiming to lower A1C as seen in Figure 2. This finding supports the use of motivational goal setting in DSME programs to improve individual biomarkers. Goal setting was utilized with the participants at this medical office during routine care and group visits. 

### Home monitoring

All participants began to monitor their blood pressure with the provided monitors. Blood pressure improved in 43% of the participants. The average blood pressure did not appreciably change, and that biomarker would perhaps have been helped by incorporating additional blood pressure education components such as salt reduction, stress reduction, and smoking cessation as these approaches were used effectively in a prior study [9]. All but three patients properly checked their blood glucose at home during the project period. Fasting glucose improved in 70% of the participants during the study period.

### Food assessment

The assessment of food in the home enabled opportunities for customized patient education. Discussions on food in the home may be fundamental in guiding the participant to improve their diet as part of their self-management of diabetes. Although in-home visits facilitate an accurate understanding of food in the home, the same understanding can be accomplished in the clinic setting by using interviewing techniques, reviewing food diaries, or allowing patients to describe what foods they have in their household.

### Study limitations

Generalization of the data is limited due to the small number of participants and the lack of a control group. As the target population was ambulatory, the barriers to signing up more participants included patients traveling, working, or having other scheduling limitations, as well as difficulties in reaching some patients. One patient stated she’d need to clean her home before visitors could come, and she was too busy to do so. The barriers to obtaining all post-intervention data included lapses in patient insurance plans, lack of transportation to appointments, and lack of coverage for a three-month A1C blood draw. One patient said it was too cold to go outside when she was urged to go to her follow-up appointment in the December-January timeframe.

The effect of the project may be understated due to the timing of the intervention, which occurred during the November-December holiday season when individuals may be more apt to consume alcohol and stray from healthy eating. The participants might have improved their clinical indicators more during a different time period.

## Conclusions

The results of this study suggest that educational programs with in-home reinforcement can improve the self-management of diabetes and lead to improvement in health indicators. The biomarkers improved in many patients after the in-home diabetes education. The most notable improvements were seen in A1C and serum glucose with 90% of the participants improving their A1C and 70% improving their serum glucose. The A1C improvement was not likely due to chance as the mean change in A1C was significant (p<0.05). While additional research is warranted, this study supports the feasibility of home-based DSME and its use as a potentially effective tool in diabetes care.

The benefits of the in-home education included personal attention in ensuring the correct use of home health monitoring devices, building self-management confidence, and identifying treatment barriers in the home that may not be easily discerned in a clinic setting. Teaching the patients in their home allowed the researchers the unique opportunity to see and discuss the actual food found in the patient’s household, as well as address in-home issues such as family support, expired glucose test strips, non-functioning glucometer or incorrect insulin storage.
